# Clinical Course and Outcome of Patients with SARS-CoV-2 Alpha Variant Infection Compared to Patients with SARS-CoV-2 Wild-Type Infection Admitted to the ICU

**DOI:** 10.3390/microorganisms9091944

**Published:** 2021-09-13

**Authors:** Jorge Garcia Borrega, Jan-Hendrik Naendrup, Katrin Heindel, Laura Hamacher, Eva Heger, Veronica Di Cristanziano, Antje-Christin Deppe, Fabian Dusse, Wolfgang Alois Wetsch, Dennis Alexander Eichenauer, Alexander Shimabukuro-Vornhagen, Boris Böll, Matthias Kochanek

**Affiliations:** 1 First Department of Internal Medicine, Center for Integrated Oncology Aachen Bonn Cologne Dusseldorf (CIO), Faculty of Medicine and University Hospital Cologne, University of Cologne, 50937 Cologne, Germany; jorge.garcia-borrega@uk-koeln.de (J.G.B.); jan-hendrik.naendrup@uk-koeln.de (J.-H.N.); katrin.heindel@uk-koeln.de (K.H.); laura.hamacher@uk-koeln.de (L.H.); dennis.eichenauer@uk-koeln.de (D.A.E.); alexander.shimabukuro-vornhagen@uk-koeln.de (A.S.-V.); boris.boell@uk-koeln.de (B.B.); 2 Institute of Virology, Faculty of Medicine and University Hospital Cologne, University of Cologne, 50937 Cologne, Germany; eva.heger@uk-koeln.de (E.H.); veronica.di-cristanziano@uk-koeln.de (V.D.C.); 3 Department of Cardiothoracic Surgery, ECMO Centre Cologne, Heart Centre, Faculty of Medicine and University Hospital of Cologne, University of Cologne, 50937 Cologne, Germany; antje-christin.deppe@uk-koeln.de; 4 Department of Anaesthesiology and Intensive Care Medicine, Faculty of Medicine and University Hospital of Cologne, University of Cologne, 50937 Cologne, Germany; fabian.dusse@uk-koeln.de (F.D.); wolfgang.wetsch@uk-koeln.de (W.A.W.)

**Keywords:** SARS-CoV-2 virus variant, Alpha variant, COVID-19, intensive care medicine, mortality

## Abstract

The alpha variant of the severe acute respiratory syndrome coronavirus type 2 (SARS-CoV-2) is associated with higher transmissibility and possibly higher mortality compared with wild-type SARS-CoV-2. However, few data are available on the clinical course of infections with the alpha variant compared with wild-type SARS-CoV-2 in critically ill patients in intensive care units (ICUs). Therefore, we retrospectively analyzed patients admitted to our ICU due to SARS-CoV-2 Alpha variant infection and compared characteristics and course to patients with SARS-CoV-2 wild-type infection. The median age of patients with Alpha variant infections was 57 years compared to 62 years in the wild-type group. ICU survival was 41/80 (51%) in the Alpha variant group and 35/80 (44%) in the wild-type group (*p* = 0.429). Results of a matched-pair analysis based on age and sex illustrated that 45/58 patients (77.6%) in the Alpha variant group and 38/58 (65.5%) patients in the wild-type group required mechanical ventilation (*p* = 0.217). ICU survival was documented for 28/58 patients (48.3%) in the Alpha variant group and 27/58 patients (46.6%) in the wild-type group (*p* = 1). Thus, ICU mortality among patients with SARS-CoV-2 infections remains high. Although the Alpha variant group included younger patients requiring mechanical ventilation, no significant differences between patients with the SARS-CoV-2 Alpha variant and the SARS-CoV-2 wild-type, respectively, were detected with respect to clinical course and ICU mortality. For future VOCs, we believe it would be important to obtain valid and rapid data on the clinical course of critically ill patients who test positive for COVID-19 in order to perform appropriate epidemiological planning of intensive care capacity.

## 1. Introduction

The ongoing global coronavirus-19 disease (COVID-19) pandemic, caused by severe acute respiratory syndrome coronavirus 2 (SARS-CoV-2) [1,2], poses major challenges to health systems worldwide. While the majority of infected people have mild to moderate symptoms, some patients develop acute respiratory distress syndrome (ARDS) requiring intensive care treatment and mechanical ventilation [3].

SARS-CoV-2 is a positive-sense single-stranded RNA virus whose genome is of a low stability and is thus more prone to mutation accumulation, with approximately 9.8 × 10^4^ substitutions/site yearly [4]. By the beginning of May 2021, there had been more than 1.4 million sequences reported, and among them, 3913 major representative variant genomes that have been identified and included in the global SARS-CoV-2 sequence database operated by Global Initiative on Sharing Avian Influenza Data (GISAID). Not all genetic mutations lead to variation in major proteins and/or alter virus infectivity. In the meantime, several mutations of SARS-CoV-2 have emerged [4]. These genetic variants affect the course of the disease by altered virulence, susceptibility to immune response and transmissibility. Four of these variants are classified as variants of concern (VOC) by the World Health Organization (WHO): Alpha variant/Lineage B.1.1.7 (first detected in the UK) [5], Beta variant/Lineage B.1.351 (first detected in South Africa) [6], Gamma variant/Lineage P.1 (first detected in Brazil) [7] and Delta variant/Lineage B.1.617.2 (first detected in India) [8]. The current spike gene mutations account for most of the clinically influential VOC. 

To date, the pandemic has evolved in waves with rapidly increasing infections and deaths in most countries. These waves led to various measures, such as lockdowns, mandatory masks and others, which consequently resulted in decreasing infection rates.

In Germany, the second COVID-19 wave in the autumn of 2020 was still caused by the SARS-CoV-2 wild-type (WT), but the Alpha variant successively superseded the WT and constituted the predominant COVID-19 pathogen from March 2021 onwards. The SARS-CoV-2 Alpha variant (lineage B.1.1.7) was first detected in the UK in September 2020 [5] and was shortly after named the Alpha variant. The Alpha variant has an N501Y mutation: at the 501 residue, asparagine N has been replaced with Y tyrosine, and K417N, lysine K, has been replaced with asparagine N. Evaluations [9] of the Robert Koch Institute illustrated a continuous increase in the proportion of infections with the Alpha variant up to more than 90% at the end of April 2021 [10]. Previous studies described the Alpha variant as being significantly more contagious. Evidence suggests that the VOC Alpha increased the transmissibility rate by ~50%, especially in younger age groups and children [11]; however, data regarding the severity of disease when compared to the SARS-CoV-2 WT are inconclusive [12,13,14,15,16,17]. At present, very limited data are available regarding the course of patients requiring admission to intensive care units (ICU) and the impact of the Alpha variant on ICU mortality [17].

Therefore, the aim of this study was to analyze the outcome and clinical course of patients with Alpha variant SARS-CoV-2 infections in the ICU of a maximum care hospital in Germany and to compare it to patients with WT infections.

## 2. Materials and Methods

Prior to the start of the study, approval was obtained by the ethics committee of the Faculty of Medicine of the University of Cologne (approval number: 20-11729). Given the non-interventional retrospective nature of the study, no informed consent needed to be obtained from the included patients.

Patients who were admitted to the ICU between 1 September 2020 and 5 May 2021 and tested positive for SARS-CoV-2 were included in the analysis. Patients were assigned to the Alpha variant or WT group if they tested positive for the SARS-CoV-2 Alpha variant B.1.1.7 (20I/501Y.V1) or SARS-CoV-2 WT, respectively. Variant identification was performed by melting curve analysis (TIB Molbiol©, Berlin, Germany). Patient characteristics and pre-existing conditions at the time of ICU admission, as well as data on the clinical course in the ICU, were extracted from the patient charts and statistically analyzed. For better comparability and to account for confounding factors, a matched-pair analysis of both groups was performed based on gender and age (±2 years). Alpha variant and WT group categorical variables were compared using Chi-square tests; continuous variables were analyzed using two-sided t-tests. Statistical significance was set to *p* < 0.05. SPSS (IBM, version 27.0.1.0, Armonk, NY, USA) and Microsoft Excel (Microsoft, version 2105, Redmond, WA, USA) were used for statistical analysis.

## 3. Results

### 3.1. Baseline Characteristics of Full Patient Population (n = 160)

One hundred and sixty patients who were admitted to our ICU were included in this analysis. Of those, 80 patients each tested positive for the Alpha variant and WT. Male patients accounted for 59 of 80 cases (74%) in the Alpha variant group and 57 of 80 cases (71%) in the WT group (*p* = 0.073). The median age was 55.5 years (range 13–83) in the Alpha variant group and 62.5 years (range 16–87) in the WT group. Despite the lower median age, the patients in the Alpha variant group had slightly higher rates for chronic respiratory and cardiovascular disease compared to the patients in the WT group. None of the differences in pre-existing conditions between the Alpha variant and the WT group reached statistical significance (Table 1).

### 3.2. ICU Procedures and Outcomes in Full Patient Population (n = 160)

In total, 61 of the 80 patients (76%) in the Alpha variant group and 52 of the 80 (65%) patients in the WT group required mechanical ventilation (MV) during their ICU stay (*p* = 0.165). The median duration of MV was 16 days (range 2–61) in the Alpha variant group and 14 days (range 1–55) in the WT group (*p* = 0.814). Among ICU survivors, the median duration of ventilation was 11 days (range 3–61) in the Alpha variant group (*n* = 23) and 12 days (range 1–55) in the WT group (*n* = 18) (*p* = 0.814). Overall, 6 of the 80 patients (8%) in the Alpha variant group and 14 of 79 patients (29%) in the WT group received extracorporeal membrane oxygenation (ECMO) therapy (*p* = 0.365). In total, 17 of 77 patients (22%) in the Alpha variant group and 23 of the 80 patients (29%) in the WT group required renal replacement therapy (RRT) (*p* = 0.365). Data regarding ECMO and RRT could not be obtained for one and three patients, respectively.

**Figure 1:**
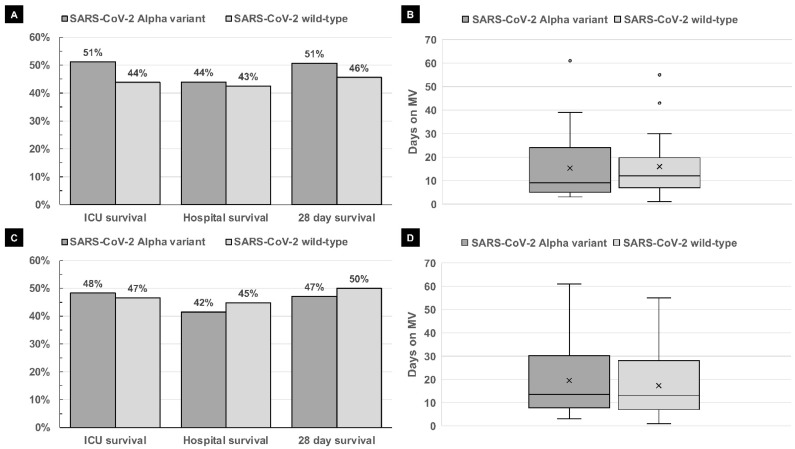
(**A**) ICU survival, hospital survival and 28-day survival in the full patient population in the SARS-CoV-2 Alpha variant group and SARS-CoV-2 wild-type group (*n* = 80 each). (**B**) Duration of mechanical ventilation (MV) in days of the entire patient population in the SARS-CoV2 Alpha group and SARS-CoV2 wild-type group (*n* = 80 each); X = mean; line inside the box marks the median; the bottom and top of the box correspond to the upper and lower quartiles, respectively; upper and lower whiskers correspond 1.5 times the interquartile range. (**C**) ICU survival, hospital survival and 28-day survival of the matched-pair analysis in the SARS-CoV-2 Alpha variant group and SARS-CoV-2 wild-type group (*n* = 58 each). (**D**) Duration of mechanical ventilation (MV) in days of the matched-pair analysis in the SARS-CoV-2 Alpha variant group and SARS-CoV-2 wild-type group (*n* = 58 each); X = mean; line inside the box marks the median; the bottom and top of the box correspond to the upper and lower quartiles, respectively; upper and lower whiskers correspond to 1.5 times the interquartile range. No significant differences were detected between the SARS-CoV-2 Alpha variant group and the SARS-CoV-2 wild-type group in any of the illustrated analyses.

Overall, 41 of the 80 patients (51%) in the Alpha variant group and 35 of the 80 patients (44%) in the WT group survived the ICU stay (*p* = 0.429). The data showed that 33 of the 75 patients (44%) in the Alpha variant group and 34 of the 80 patients (43%) in the WT group survived the hospital stay (*p* = 0.872); no data regarding the hospital survival could be obtained for five patients in the Alpha variant group. In total, 36 of 71 patients (51%) in the Alpha variant group and 36 of 79 patients (46%) in the WT group were still alive 28 days after initial admission to the ICU (*p* = 0.624); no data on 28-day survival could be obtained for nine patients in the Alpha variant group and one patient in the WT group. Table 2 and Figure 1 show the data regarding the ICU stay and outcomes in the Alpha variant and WT group, both for the full patient population and the matched-pair analysis.

### 3.3. Baseline Characteristics of the Matched-Pair Analysis (n = 116)

Using gender and age (±2 years) as matching criteria, a total of 116 patients were included in the matched-pair analysis. The median age of the patients was 61 years (range 25–85) in the Alpha variant group and 62 years (range 26–83) in the WT group. In both groups, 43 of the 58 patients (74%) were male. The patients from the Alpha variant had higher rates for diabetes and chronic respiratory diseases, while the patients from the WT group had a higher rate for oncologic, hematologic or immunosuppressive conditions. None of the differences in pre-existing conditions between the two groups reached statistical significance. Table 1 summarizes the demographic data and pre-existing conditions for patients from both groups.

### 3.4. ICU Procedures and Outcomes of the Matched-Pair Analysis (n = 116)

A total of 45 of the 58 patients (78%) from the Alpha variant group and 38 of the 58 patients (66%) from the WT group required MV during their ICU stay (*p* = 0.217). The median duration of ventilation was 16 days (range 2–61) in the Alpha variant group and 14.5 days (range 1–55) in the WT group (*p* = 0.694). Among patients who survived the ICU stay, the median duration of ventilation in the Alpha variant group (*n* = 28) was 13.5 (range 3–61) and 13 days (range 1–55) in the WT group (*n* = 15) (*p* = 0.694). In total, 10 of the 58 patients (23%) in the Alpha variant group and 6 of the 58 patients (10.3%) in the WT group required ECMO therapy (*p* = 0.420). A total of 13 of 56 patients (23%) in the Alpha variant group and 17 of the 58 patients (29%) in the WT group required RRT (*p* = 0.526). Data regarding RRT could not be obtained for two patients in the Alpha variant group.

Overall, 28 of the 58 patients (48%) in the Alpha variant group and 27 of the 58 patients (47%) in the WT group survived the ICU stay (*p* = 1); 22 of 53 patients (42%) in the Alpha variant group and 26 of the 58 patients (45%) in the WT group survived the hospital stay (*p* = 0.848). No data on hospital survival were available for five patients in the Alpha variant group. Overall, 24 of 51 patients (47%) in the Alpha variant group and 29 of the 58 patients (50%) in the WT group were alive 28 days after initial ICU admission (*p* = 0.848); no data on 28-day survival were available for seven patients in the Alpha variant group. Table 2 and Figure 1 illustrate the outcomes of the Alpha variant and WT groups.

## 4. Discussion

The Alpha variant is estimated to have emerged in January 2021 and has quickly become the dominant circulating SARS-CoV-2 variant in Germany. The Alpha variant has been detected all over the world. Multiple lines of evidence indicate that the Alpha variant is more efficiently transmitted than other SARS-CoV-2 variants. Currently, there is scarce knowledge about the differences in the clinical outcomes of the SARS-CoV-2 variants described; however, a higher rate of transmission will result in more cases, increasing the overall number of people requiring clinical care, increasing the burden on the already strained health care system, and resulting in more deaths. The risk of hospital admission is higher for people infected with the Alpha variant compared with wild-type SARS-CoV-2, likely reflecting a more severe disease [18]. In a study from the UK, a dataset of over 2.2 million positive SARS-CoV tests was evaluated for mortality. The corrected risk of death in this large cohort was 61% higher for patients infected with the Alpha variant than with the wild type. However, all patients were studied in this cohort. Detailed evaluations of clinical courses of critically ill patients in ICUs were not performed [12]. 

Until now, data regarding the intensive care treatment of patients with SARS-CoV-2 Alpha variant infection and SARS-CoV-2 WT infection, respectively, have been limited, and initial clinical impressions of critically ill patients with the Alpha variant did not yield consistent data regarding clinical courses and outcome. The data of this study are the first data from clinical courses of critically ill patients with the Alpha variant so far. The results of the present study indicate that critically ill patients in intensive care units with severe COVID-19 infection caused by the SARS-CoV-2 Alpha variant were on average 7 years younger than patients with SARS-CoV-2 WT infection. When analyzing the whole patient cohort, the ICU survival was slightly higher in patients with the SARS-CoV-2 Alpha variant compared to the SARS-CoV-2 WT group, despite an increased number of patients requiring mechanical ventilation. The difference in the ICU survival could be caused by the younger patients included in the Alpha variant group, especially since this slight and non-significant difference was not seen in a matched-pair analysis accounting for the confounding factors of gender and age. Here, survival was almost identical in both groups. The increased mortality of the WT group measured in the entire patient population is, therefore, most likely attributable to the higher age in the WT group. As of 12 May 2021, a total of 36,837,184 vaccinations had been administered in Germany. Moreover, 10% had already been fully vaccinated against COVID-19 [19]. We believe that the onset of vaccination changed the average age of patients in the ICU during the study period but did not affect the course of the disease itself. This may not be true for future studies. 

Whether there was an influence of vaccination on the results of this evaluation can only be conjectured. During the study period from September 2020 to May 2021, none of the patients studied here had received one or two vaccinations. 

While some previous cohort studies showed an increased mortality in patients with SARS-CoV-2 Alpha variant infections [12,13,14], the present study including only critically ill patients could not confirm this result. Several factors might contribute to the different findings, e.g., structural differences within the health systems but also different age distributions and ethnicities of patients included in the different analyses [15]. Our present data are more in line with recently published results from Spain where the Alpha variant infected younger patients, and led to an increase in ICU admissions but not to an increased mortality when compared to patients with SARS-CoV-2 WT infection [17].

Even though previous studies had a significantly higher number of patients, no analysis restricted to the course of ICU patients had been undertaken. This fact hampers a direct comparison.

Patients were only included in the WT group if the diagnosis was made after 1 September 2020, as treatment of COVID-19 in ICUs had become more standardized compared to the beginning of the pandemic in spring 2020. A number of national and international guidelines have been produced with sufficient quality of evidence [20]. In particular, therapy with dexamethasone was established as a standard of care, resulting in a significant survival benefit [21]. 

In our opinion, there is probably no relevant difference in the clinical course of critically ill COVID-19 patients in the ICU related to the variant of SARS-CoV-2 when they have similar age and sex. The clinical picture from practice sometimes leads one to believe otherwise.

The present study has limitations associated with its retrospective, monocentric study design. In particular, the selected patient cohort at the reporting university clinic as a tertiary care hospital increases the risk of selection bias. Although the city of Cologne’s emergency care plan has distributed patients with COVID-19 evenly across all city hospitals, critically ill and even younger patients are more likely to be admitted primarily or secondarily to a maximum care hospital. Despite the evaluation of a total of 160 patients and 116 patients in the matched-pair analysis, only a tendentious impression of the clinical course of the SARS-CoV-2 Alpha variant in ICUs could be given. Due to the uneven and rapidly changing distributions of virus variants, we were only able to examine one variant in comparison to the SARS-CoV-2 WT in this present evaluation. For comparison with other virus variants, a multicenter study would have to be performed due to the limited number of cases. From our perspective, therefore, the rapid evaluation of variant trajectories is extremely important for pandemic hospital planning in the intensive care setting. Large multicenter registry databases certainly provide more valid information compared to the single-center study conducted here; however, the results are usually published with a significant delay.

## 5. Conclusions

In summary, mortality among patients with severe COVID-19 treated in the ICU remains high. Recently, more younger patients have required intensive care treatment. Whether the reasons for this development are changes in the transmissibility due to viral variants, the advancing vaccination campaign, and/or the virus spread in a younger segment of the population remains uncertain. At present, the impact of SARS-CoV-2 variants on the severity of the disease as well as on ICU mortality has not been elucidated. However, in the present age- and sex-matched analysis, no significant differences with respect to clinical course and mortality in intensive care units between the SARS-CoV-2 Alpha variant and the SARS -CoV-2 WT were detected. For future VOCs, we believe it would be important to obtain valid and rapid data on the clinical course of critically ill patients who test positive for COVID-19 in order to perform appropriate epidemiological planning of intensive care capacity.

## Figures and Tables

**Table 1:** Baseline patient characteristics of both the full patient population as well as the matched-pair analysis. No significant differences were identified between the SARS-CoV2 Alpha variant group and the SARS-CoV2 wild-type group. #: matching criteria (sex, age).

	Full Patient Population (*n* = 160)			Matched-Pair Analysis (*n* = 116)		
	Alpha Variant (*n* = 80)	Wild Type (*n* = 80)	*p*	Alpha Variant (*n* = 58)	Wild Type (*n* = 58)	*p*
Age (median, range)	55.5 (13–83)	62.5 (16–87)	0.073	61 (26–83) #	62 (25–85) #	0.917
Males	59/80 (74%)	57/80 (71%)	0.860	43/58 (74.1%) #	43/58 (74.1%) #	1
Diabetes	25/80 (26%)	21/80 (31%)	0.601	20/58 (34.5%)	14/58 (24.1%)	0.308
Chronic respiratory diseases	13/80 (16%)	10/80 (13%)	0.653	9/58 (15.5%)	4/58 (6.9%)	0.238
Cardiovascular disease	44/80 (55%)	40/80 (50%)	0.635	30/58 (51.7%)	30/58 (51.7%)	1
Oncologic/hematologic disease/immunosuppressive medication	11/80 (14%)	16/80 (20%)	0.399	9/58 (15.5%)	12/58 (20.7%)	0.630
Liver disease	8/80 (10%)	4/80 (5%)	0.369	5/58 (8.6%)	3/58 (5.2%)	0.717

**Table 2:** Outcome and ICU-specific therapies both in the full patient population as well as in the matched-pair analysis (sex, age). No significant differences were identified between the SARS-CoV2 Alpha variant group and the SARS-CoV2 wild-type group. MV = mechanical ventilation; NIV = non-invasive ventilation; HFNO = high-flow nasal oxygen.

	Full Patient Population (*n* = 160)			Matched-Pair Analysis (*n* = 116)		
	Alpha (*n* = 80)	Wild Type (*n* = 80)	*p*	Alpha (*n* = 58)	Wild Type (*n* = 58)	*p*
ICU survival	41/80 (51%)	35/80 (44%)	0.429	28/58 (48%)	27/58 (47%)	1
Hospital survival	33/75 (44%)	34/80 (43%)	0.872	22/53 (42%)	26/58 (45%)	0.848
28-day survival	36/71 (51%)	36/79 (46%)	0.624	24/51 (47.1%)	29/58 (50%)	0.848
Mechanical ventilation	61/80 (76%)	52/80 (65%)	0.165	45/58 (78%)	38/58 (66%)	0.217
Duration of mechanical ventilation (days) (median, range)	16 (2–61)	14 (1–55)	0.814	16 (2–61)	14.5 (1–55)	0.694
Duration of mechanical ventilation of ICU survivors (days) (median, range)	11 (3–61) [*n* = 23]	12 (1–55) [*n* = 18]	0.814	13.5 (3–61) [*n* = 28]	13 (1–55) [*n* = 15]	0.694
Duration of NIV/HFNO in patients with no MV (days) (median, range)	6 (2–14) [*n*= 16]	5.5 (1–20) [*n* = 16]	0.934	5 (2–9) [*n* = 11]	4 (1–13) [*n* = 11]	0.844
ECMO	6/80 (8%)	14/79 (18%)	0.059	10/58 (17%)	6/58 (10%)	0.420
Renal replacement therapy	17/77 (22%)	23/80 (29%)	0.365	13/56 (23%)	17/58 (29%)	0.526

## Data Availability

The data presented in this study are available on request from the corresponding author.
